# Visualizing neutrophil extracellular traps in septic equine synovial and peritoneal fluid samples using immunofluorescence microscopy

**DOI:** 10.1177/10406387231196552

**Published:** 2023-09-03

**Authors:** Emily M. Birckhead, Shubhagata Das, Naomie Tidd, Sharanne L. Raidal, Shane R. Raidal

**Affiliations:** School of Agricultural, Environmental and Veterinary Sciences, Faculty of Science and Health, Charles Sturt University, Wagga Wagga, NSW, Australia; School of Agricultural, Environmental and Veterinary Sciences, Faculty of Science and Health, Charles Sturt University, Wagga Wagga, NSW, Australia; Veterinary Diagnostic Laboratory, Charles Sturt University, Wagga Wagga, NSW, Australia; School of Agricultural, Environmental and Veterinary Sciences, Faculty of Science and Health, Charles Sturt University, Wagga Wagga, NSW, Australia; School of Agricultural, Environmental and Veterinary Sciences, Faculty of Science and Health, Charles Sturt University, Wagga Wagga, NSW, Australia

**Keywords:** histones, horses, immunofluorescence, infection, NETs, peritoneal fluid, synovial fluid

## Abstract

Septic synovitis and peritonitis are routinely diagnosed in horses based on clinical examination findings and laboratory assessment of synoviocentesis and abdominocentesis samples, respectively. Diagnosis is difficult in some cases because of an overlap in laboratory results for septic and non-septic inflammation. Neutrophil extracellular trap (NET) formation is part of the innate immune response against pathogens. Identifying and quantifying NETs, which have not been explored in clinical samples from horses with septic synovitis and peritonitis, to our knowledge, may be helpful in detecting infectious processes. Our main objective was to determine whether NETs could be visualized in septic equine synovial and peritoneal fluid cytology samples using immunofluorescence with antibodies against citrullinated histone H3 (Cit-H3) and myeloperoxidase (MPO). We analyzed 9 synovial and 4 peritoneal fluid samples. NET percentages were quantified using a simple counting technique, which is suitable for high-quality, well-preserved, and stained cytospin smears. NETs were evident in all septic samples and were absent in a non-septic sample; NETs were better visualized with Cit-H3 than with MPO immunolabeling. Overall, we believe that there is the potential for NETs and associated markers to be used to investigate and understand septic inflammation in horses.

Neutrophil extracellular trap (NET) formation is an innate immune defense mechanism against pathogens. It is well conserved among animals, although most studies have involved humans and mice, and NET formation in horses has, to date, been poorly characterized. Various stimuli can induce NETs, including infectious organisms, biological molecules, and chemicals.^49^ Isolated equine neutrophils have released NETs when exposed to *Streptococcus equi* subsp. *zooepidemicus*, *Staphylococcus capitis*, or *Escherichia coli*,^56^ to equine cathelicidins,^21^ and the chemical phorbol–myristate–acetate.^56^ NETs are comprised of extruded DNA and associated antimicrobial proteins, including histones and granule contents, and can entrap and potentially kill microbes.^49^ NETs can also help with the resolution of inflammation, particularly at higher neutrophil densities, by forming large NET aggregates and degrading inflammatory mediators.^27^ However, when this mechanism is out of balance, as a result of excessive formation or inadequate clearing of NETs, there is an uncontrolled release of proteases and production of reactive oxygen species, which can cause tissue damage.^61^ NET release can cause necrosis or apoptosis of various cells, including endothelium,^26,58^ alveolar epithelium,^58^ intestinal epithelium,^66^ and synoviocytes.^35^ Large aggregates of NETs can obstruct vessels and ducts.^40,61^

Increased NET release has been detected in various infectious conditions. In veterinary studies, this has included dogs with sepsis,^41^ and dogs and cats with pyometra.^55^ In horses, there have been limited studies on NETs and their involvement in infectious diseases. NETs have been detected in uterine samples from mares with endometritis.^56^ Other researchers found that plasma nucleosome levels, a NET marker, were increased significantly in horses with inflammatory or strangulating gastrointestinal disease.^3^ Similarly, plasma cell–free DNA, a marker that can also be increased as a result of NET release, was elevated in equine colic patients.^4^ However, the presence of infectious processes was not confirmed in these studies.^3,4^ Interestingly, plasma cell–free DNA concentrations were not elevated in septic foals.^16^ Non-infectious and immune-mediated diseases can also be associated with NET release,^14^ and in horses this has included equine asthma^32,72^ and recurrent uveitis.^21,22^

The presence of NETs in septic equine synovial fluid (SF) and peritoneal fluid (PF) samples is unknown, and therefore it is unclear if those fluids could be of use in the diagnosis of infection or septic inflammation and for predicting prognosis. Diagnosis of septic inflammation can be challenging using routine laboratory methods given overlapping results for non-septic and septic processes. Bacterial culture is slow and can result in false negatives, and cytology has poor sensitivity for detecting infection.^1,2,28,64^ There is also a delay in normalization of laboratory results despite resolution of infection; this has been shown for neutrophil percentage and total protein (TP) in SF samples,^38^ and serum amyloid A in horses with synovial infection.^75^

Myeloperoxidase (MPO), which assists with the unfolding of chromatin and forms a major component of NETs,^66,76^ has been studied in SF and PF samples of horses. Septic SF had significantly elevated MPO compared to aseptic synovitis and healthy control samples,^28^ and MPO was also higher in PF from horses with necrotic intestines.^25^ But MPO is not specific for NETs; it is normally present in resting neutrophils,^54^ and can be increased with neutrophil degranulation and necrosis.^28,42^ Evaluation of septic peritoneal effusions from dogs has demonstrated increased NET markers,^46^ and NETs were visualized with immunofluorescence.^42^ We retrieved no cases of septic SF samples containing NETs in a search of Google Scholar, PubMed, CAB Abstracts, Web of Science, and Scopus, using the search terms: neutrophil extracellular traps, synovial fluid, synovitis, septic, infection, veterinary, canine, feline, ruminant, suggesting that this condition has not been reported in other veterinary species. In human studies, NET markers in SF were significantly increased in cases of septic arthritis compared to non-septic joint inflammation and osteoarthritis,^45^ and also in people with chronic periprosthetic joint infection.^15^

Direct visualization and quantification of NET release have been recommended as the gold standard method for NET assessment.^17^ NETs can be visualized with immunofluorescence, using antibodies targeting specific proteins that make up NETs, including citrullinated histone 3 (Cit-H3) and MPO, and with concurrent staining of DNA.^32,42,65^ Citrullination of histones by the enzyme peptidylarginine deiminase 4 is highly correlated with NET release^76^; however, citrullination does not always occur with NET formation and can be associated with other forms of cell death and with different cells.^24,76^ Therefore, using a second marker with Cit-H3, such as MPO or neutrophil elastase, has been recommended to increase the reliability of results.^76^ Visualizing extracellular filaments with co-localization of these NET components is a strong indicator of NET formation.^32^

Our primary objective was to determine if NETs can be identified in SF and PF samples from horses with septic synovitis and septic peritonitis. Our hypotheses were that there would be NETs in septic samples and that NET formation would be greater in septic samples than in non-septic samples. A secondary objective was to compare the appearance of NETs in SF and PF using antibodies directed against Cit-H3 and MPO.

## Materials and methods

### Samples

We analyzed septic and non-septic equine SF and PF fluid samples submitted to the Veterinary Diagnostic Laboratory (VDL), Charles Sturt University (CSU, Wagga Wagga, New South Wales, Australia), in May–November 2021. All samples were from horses being treated at the Veterinary Clinical Centre, CSU. We included samples submitted for diagnostic purposes if there was surplus material, and therefore ethics approval was not required. Full histories and clinical data, including SF and PF total nucleated cell counts (TNCC), cytology findings, and microbial culture results, were obtained from patient records. For TNCC, SF and PF samples were diluted 1:5–1:100 in a diluent (Cellpack DCL; Sysmex), depending on cellularity. Automatic counts were performed (XN-1000 analyzer; Sysmex). If the fluid was highly viscous or contained large amounts of blood, manual counts were performed by experienced technical officers using Kova counting chambers (ELITech). TP was measured with a refractometer. Direct and cytocentrifuge smears were prepared for cytologic evaluation. SF and PF samples were added to cytospin funnels in 10–50-μL or 5–80-μL aliquots, respectively, with smaller volumes used for highly cellular samples, and centrifuged at 28 × *g* for 5 min (Cytospin 3 cytocentrifuge; Thermo Shandon). Slides were stained (Hematek automated slide stainer; Siemens) with Wright–Giemsa stain (Hematek stain pack; Siemens). Each smear was examined, and a 100-cell differential was performed by a veterinary pathologist and/or pathology resident.

As reported previously,^63,64^ a diagnosis of septic synovitis was based on meeting at least one of the following criteria: TNCC of > 30 × 10^9^ cells/L, TP > 40 g/L, neutrophils > 80% of differential cell count, a positive bacterial culture of SF, or intracellular bacteria present on cytologic examination of SF. For PF analysis, a TNCC ≥ 20 × 10^9^ cells/L, TP > 30 g/L, and the presence of bacteria on cytology and/or bacterial growth on culture of PF were diagnostic for septic peritonitis.^2,53^ Negative controls included samples with TNCC and TP within the RIs used at the VDL (i.e., peritoneal TNCC < 5 × 10^9^ cells/L, TP < 25 g/L; synovial TNCC < 0.5 × 10^9^ cells/L, TP < 20 g/L). Secondary antibody controls (elimination of primary antibodies) and labeling controls (elimination of all antibodies and dyes) were performed to evaluate nonspecific binding and autofluorescence (Suppl. Fig. 1).^8^

Cytospin preparations of SF and PF samples submitted in EDTA tubes were made for immunofluorescence analysis. Cytospins were made within 3–24 h of sample collection (Suppl. Table 1), at the same time as routine tests, or from refrigerated stored samples. Samples were stored at room temperature if processing was within 4 h of collection, or kept at 4°C if processing was delayed. We prepared 2–4 cytospin preparations from each sample, depending on the amount of fluid available (Cytospin 3 cytocentrifuge; 28 × *g* for 5 min), as described previously. Slides were air-dried and then frozen at −60°C in slide holder containers enclosed in resealable bags until immunofluorescence analysis 101–289 d later.^20,52,62^

### Immunofluorescence staining and analysis

Cytospin slides were defrosted at room temperature immediately before immunofluorescence analysis. A Pap pen was used to create a hydrophobic barrier around the cytospin samples to contain reagents and antibodies for incubation steps and minimize required volumes. Typically, 100 μL/slide was adequate to cover cytology specimens. Fixing and washing steps involved soaking slides within solution-filled Coplin jars. Incubation steps were carried out within plastic slide-staining boxes with secured lids. All steps were performed at room temperature.

The primary antibodies were a rabbit polyclonal antibody against MPO (ab9535; Abcam) and polyclonal and monoclonal rabbit antibodies against Cit-H3 (ab5103, ab219407; Abcam, respectively), all diluted at 1:200 (DM830 antibody diluent; Dako). We chose a monoclonal antibody for Cit-H3 fluorescence because of the greater specificity it may offer, with fewer off-target interactions compared to a polyclonal antibody. Monoclonal antibodies are generated from one clone of B cells, whereas polyclonal preparations consist of a heterogeneous group of antibodies produced by different B cells.^11^ The secondary antibody was a polyclonal goat anti-rabbit IgG antibody conjugated to Alexa Fluor 488 (ab150077; Abcam), diluted at 1:1,000 (DM830 antibody diluent; Dako). Separate cytospin preparations were used for each primary antibody; each sample was stained for MPO and Cit-H3 (monoclonal antibody), and a proportion were stained using the polyclonal Cit-H3 for comparison purposes. All antibodies were from single lots to minimize variation.

The immunofluorescence protocol was based on a previous study, with modifications.^72^ Cytospin smears were fixed in 3% paraformaldehyde in PBS for 3–4 min, followed by 3–4 washes in PBS. Smears were incubated in 0.1% Triton X-100 in PBS for 15 min, then washed in PBS 3 times. Bovine serum albumin was applied; samples were incubated for 15 min and then washed once in PBS. Slides were incubated in the primary antibody for 1.5 h, and then the secondary antibody for 1 h, protected from light. Slides were washed 3 times in PBS between application of the primary and secondary antibodies, and again after incubation with the secondary antibody. A drop of Fluoroshield with mounting medium (DAPI F6057; Sigma-Aldrich), for DNA staining,^42^ was applied to coverslips, placed onto samples, and allowed to incubate for ≥ 5 min before microscopic examination. Coverslips were sealed with clear nail polish, and slides were stored in an enclosed dark box in a cold-room if there was any delay in microscopic examination, or in a slide container at −60°C if examination did not occur within 24 h.

Slides were examined with a fluorescence microscope (BX53 microscope; Olympus), using fluorescein isothiocyanate (FITC) and 4′,6-diamidino-2-phenylindole (DAPI) filters for visualization of primary antibodies and DNA, respectively. Images were captured (Cell Sense standard software; Olympus). Exposure times were manually adjusted to optimize immunofluorescence quality against a black background for both DAPI and FITC. Image merging was performed using Cell Sense standard software, and brightness and contrast were adjusted as necessary. For NET counting, images were uploaded to ENVI 5.6.1 (Harris Geospatial Solutions). All nuclei or DAPI-positive material appearing to be cells were counted in ≥ 4 DAPI images captured at 400× magnification (Suppl. Fig. 2). A minimum of 700 cells per sample were counted by the primary author (E.M. Birckhead). At least one image from each edge of the cytospin smear, considered to be representative of the sample based on initial microscopic analysis, was used for counting. For the FITC images, the monoclonal Cit-H3 antibody was used to identify NETs. The polyclonal Cit-H3 antibody was only used for NET counts when comparisons of slides stained with monoclonal or polyclonal Cit-H3 in the same immunofluorescent run were being made. NETS were recognized when Cit-H3–positive material was associated with extracellular DAPI-positive DNA, extending from nuclei, cell remnants, or as released fibers. NETS were also counted when Cit-H3 was localized to swollen or rounded nuclei, which occurs with early NET formation before nuclear rupture and extracellular release of DNA.^12,70^ However, it is a feature that may not be captured if DNA release occurs quickly as a result of neutrophils being exposed to potent stimuli.^19^ Faded or nonspecific fluorescence was not counted. The NET count was expressed as a percentage of all cells (nuclei).

## Results

### Animal and sample details

We analyzed samples from 11 horses, including SF from 7 and PF from 4. The horses varied in age, and consisted of 3 foals (< 3-wk-old) and 2 immature (1–3-y-old) and 6 mature (4–15-y-old) horses. Horses 2, 7, and 9 were euthanized because of a guarded prognosis and/or associated treatment costs. The remaining patients were discharged from hospital (Suppl. Table 2).

We included 9 SF samples from 7 horses. Horses 1 and 4 had SF collected from 2 separate sites. We classified 7 of 9 SF samples as septic; 6 samples met ≥ 2 inclusion criteria for septic inflammation (Table 1). Six samples had a TNCC > 30 × 10^9^ cells/L, and 5 of these had a TP >40 g/L and/or > 80% neutrophils on differential cell count. Microbial culture was performed for 8 of 9 SF samples, and 4 samples (50%) were positive for bacterial growth (Suppl. Table 3). Four horses received antimicrobial treatment within 3 d of sample collection for culture (Table 1). Bacteria were not detected on cytologic examination of any SF samples. Two SF samples, both from horse 1, did not meet the septic inflammation inclusion criteria. The tarsocrural joint of this horse was open (breached), and the SF evidenced moderate suppurative inflammation but did not meet diagnostic cutoffs. Another SF sample from the radiocarpal joint of this horse had increased TNCC and TP consistent with mild inflammation. RBC counts were low (≤ 15.0 × 10^9^/L) in all of the analyzed SF, apart from 2 samples (horse 4; Suppl. Table 3). The low RBC counts were consistent with iatrogenic hemorrhage during sample collection. The higher RBC count samples may have reflected some degree of acute pre-sampling hemorrhage associated with inflammation and/or trauma.

**Table 1.** Analysis of synovial fluid samples from 7 horses using routine laboratory tests and immunofluorescence to identify and count neutrophil extracellular traps.

Horse	Sample site	TNCC/TP	NEUT, %	Bacterial growth	NET count	NET, %
1	RCJ	1.1/30	7	N	NA	NA
	TCJ†	16.2/20	76	NA	10/1,014	1.0
2*	DIPJ†	212/50	97	Y	4/1,255	0.3
3*	MTPJ	36/46	67	Y	7/1,196	0.6
4*	LFTJ†	57/52	96	N	61/815	7.5
	MFTJ†	61/40	93	N	NA	NA
5*	TCJ‡	26/30	96	Y	6/1,047	0.6
6*	TCJ†	37/36	61	N	8/1,049	0.8
7*	BB†	168/46	99	Y	27/722	3.7

BB = bicipital bursa; DIPJ = distal interphalangeal joint; LFTJ = lateral femorotibial joint; MFTJ = medial femorotibial joint; MTPJ = metatarsophalangeal joint; N = no; NA = not applicable (not performed); NET = neutrophil extracellular trap; NET count = number of NETs divided by number of cells (nuclei); NEUT = neutrophils (differential count); RCJ = radiocarpal joint; TCJ = tarsocrural joint; TNCC = total nucleated cell count (× 10^9^/L); TP = total protein (g/L); Y = yes.

* Horses with synovial fluid samples meeting inclusion criteria for septic inflammation.

† History of antibiotic treatment ≤ 3 d before sample collection.

‡ Antibiotic history unclear.

Of the PF samples, 2 of 4 were classified as septic. The septic peritonitis cases had positive microbial culture results, and bacteria were visualized during cytologic assessment of PF from 1 of the 2 horses (Table 2; Suppl. Table 3). Neither of these horses was being treated with antibiotics at the time of sample collection. The other 2 PF samples had TNCC and TP within RIs, with no cytologic evidence of infection or inflammation, and hence did not meet inclusion criteria for septic inflammation. RBC counts were low (≤ 20.0 × 10^9^/L) in all PF samples, apart from horse 9, which had septic inflammation and likely acute pre-sampling hemorrhage associated with inflammation and trauma.

**Table 2.** Analysis of peritoneal fluid samples from 4 horses using routine laboratory tests and immunofluorescence to identify and count neutrophil extracellular traps.

Horse	TNCC/TP	NEUT, %	Bacteria, cytology	Bacterial growth	NET count	NET, %
8	2.3/9	80	N	NA	0/1,068	0
9*	180/47	94	Y	Y	18/1,215	1.5
10†	3.3/11	85	N	NA	NA	NA
11*	221/63	88	N	Y	NA	NA

N = no; NA = not applicable (not performed); NET = neutrophil extracellular trap; NET count = number of NETs divided by number of cells (nuclei); NEUT = neutrophils (differential count); TNCC = total nucleated cell count (× 10^9^/L); TP = total protein (g/L); Y = yes.

* Horses with peritoneal fluid samples meeting inclusion criteria for septic inflammation.

† History of antibiotic treatment ≤ 3 days of sample collection.

### Immunofluorescence analysis

**Figure 1.**
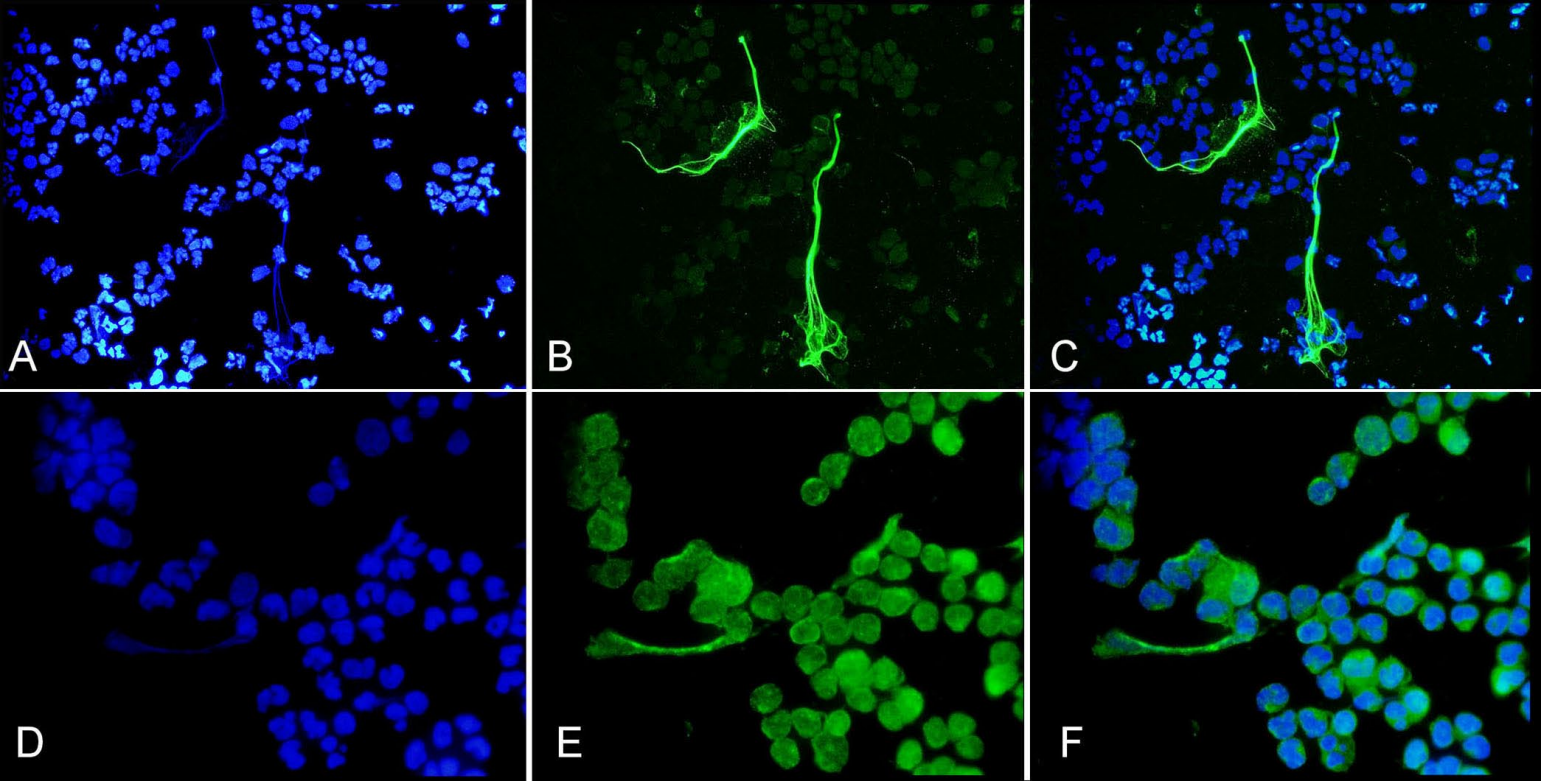
Immunofluorescent images of synovial fluid cytospins prepared from horse 2 with a septic distal interphalangeal joint. The blue DAPI filter (**A, D**) was used to detect DNA, and the green FITC filter to visualize citrullinated histone 3 (Cit-H3, monoclonal antibody; **B**) and myeloperoxidase (MPO; **E**). Co-localization of extracellular DNA fibers with Cit-H3 or MPO, evident in the merged images (**C, F**, respectively) were consistent with neutrophil extracellular traps. 400× (A–C); 1,000× (D–F).

**Figure 2.**
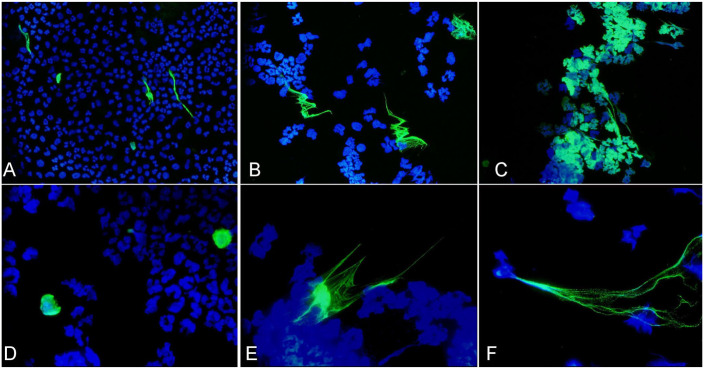
Neutrophil extracellular traps (NETs) were visualized in cytospins of septic synovial and peritoneal fluid samples from horses using immunofluorescence. A polyclonal (**A, B**) and monoclonal (**C–F**) antibody was directed against citrullinated histone 3 (Cit-H3, green), and DAPI was used to stain DNA (blue). The images have all been merged. Cit-H3 immunofluorescence staining was similar for both antibodies and varied from being localized to swollen nuclei, consistent with early NET formation (**D**), to forming slender extracellular fibers (**A, B, E, F**) or NET aggregates (**C**). Images were of synovial fluid from horses 6 (A, B, F), 7 (D), and 5 (E), and peritoneal fluid from horse 9 (C). 400× (A–C); 1,000× (D–F).

NETs were identified with antibodies targeting Cit-H3 in septic samples, and the appearance was similar in both SF and PF. The Cit-H3 immunofluorescence was readily detected at a magnification of 400×. NET morphology was variable (Figs. 1, 2; Suppl. Fig. 3), and it was difficult to predict if DNA (DAPI-positive) structures resembling NETs were true NETs or from lysed cells, given that cytologically they appear similar.^33^ Once the FITC filter was applied and Cit-H3 was visualized, only a small proportion of the structures were true NETs. Fluorescence was sometimes localized to individual swollen rounded nuclei (Fig. 2D) and had similar morphology to the NET-prone “primed” neutrophils described in an equine study.^32^ There were NETs consisting of well-defined short-to-long (up to ~160 µm) individual fibers, and with a similar appearance to spread NETs. Spread NETs are made up of fine, smooth fibers and form long web-like structures (most evident in Fig. 2F).^59^ There appeared to be aggregated NETs forming large clusters,^59^ mostly in the septic peritoneal effusion (Fig. 2C; Suppl. Fig. 4C). There was weak Cit-H3 immunofluorescence associated with nuclei, evident only with higher exposure times and magnifications.

Polyclonal and monoclonal Cit-H3 antibodies were compared using duplicate samples from horses 1 (tarsocrural joint), 2, 6, 7, and 9. Immunofluorescence staining of NETs was similar for both antibodies (Suppl. Fig. 4). A NET count was carried out using SF samples from horse 2 stained in the same run with polyclonal or monoclonal Cit-H3 antibodies. The count was the same for both antibodies, with NETs comprising 0.3% of nucleated cells.

**Figure 3.**
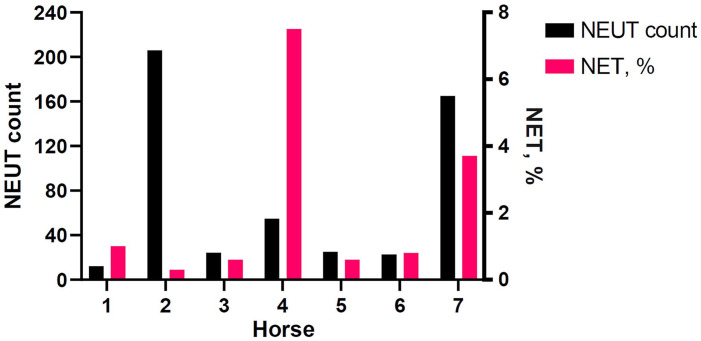
Comparison of neutrophil (NEUT) counts (× 10^9^/L) with neutrophil extracellular trap (NET) counts as percentages in synovial fluid (SF) samples from 7 horses. Neutrophil counts were calculated from the total nucleated cell count of the SF samples multiplied by the differential cell count for neutrophils. The numbers on the x-axis are the horse IDs. All samples met septic inflammation criteria, apart from horse 1 (tarsocrural joint), which had moderate suppurative inflammation.

In septic SF fluid samples, NET count percentages were 0.3–7.5% of nucleated cells (Table 1). Horse 1 (tarsocrural joint), with moderate suppurative synovial inflammation and not meeting criteria for septic inflammation, had 1% NETs. NET count percentages varied in relation to neutrophil counts and were not correlated (Fig. 3). Two SF samples were excluded from NET counts because of low cellularity and inability to count 700 cells (horse 1, radiocarpal joint), and poor cell preservation (horse 4, medial femorotibial joint). The FITC fluorescence associated with Cit-H3 was weak in the sample from horse 1, and only small NET-like structures were present in very low numbers. The cells in the poorly preserved sample (horse 4) often appeared ruptured.

NET counts were performed on 2 PF samples, including one with septic inflammation (horse 9) and one without evidence of inflammation or infection (horse 8). The NET count was 1.5% in the septic sample. No NETs were observed in the sample from horse 8. Two PF samples were excluded because of low cellularity and inability to count 700 cells (horse 10), and poor cell preservation (horse 11). The low cellularity sample only had rare FITC fluorescence (anti–Cit-H3 antibody) and did not contain distinct NETs. The poorly preserved sample had thick aggregates of cell material with more generalized FITC fluorescence, making counting and NET identification difficult. NETs were identified when a better-preserved slide replicate from horse 11 was stained with the polyclonal Cit-H3 antibody.

MPO immunofluorescence was less distinct compared to Cit-H3 (monoclonal and polyclonal antibodies), and often required 1,000× magnification for visualization (Fig. 1; Suppl. Fig. 5). MPO staining was present throughout the cytoplasm and around nuclei, and with small amounts co-localized with extracellular DNA strands. The fluorescence was inconsistently more intense on cytoplasmic edges and associated with NETs.

## Discussion

We identified NETs in septic SF samples in foals and adult horses, and also in a septic PF sample from an adult horse. NETs were more distinct with Cit-H3 immunofluorescence compared to MPO. Although the polyclonal Cit-H3 antibody that we used (ab5103) has been used in human^36,67,69^ and animal NET studies,^30,42,43^ including equine bronchoalveolar lavage (BAL) samples,^32^ our understanding is that the monoclonal Cit-H3 antibody (ab219407) has not been used in equine studies. NETs were visualized equally well using monoclonal and polyclonal antibodies. The NETs made up 0.3–7.5% of nucleated cells in the septic fluid samples, similar to a canine study.^42^ Comparison between septic and non-septic samples was limited in our study by the low number of non-septic samples. NETs were absent in a non-septic PF sample, and one SF sample that did not meet the sepsis inclusion criteria (horse 1, tarsocrural joint) had a similar NET percentage to septic inflammation samples. This horse had moderate suppurative inflammation and a synovial breach, and therefore the joint may have been septic despite not meeting diagnostic criteria for our study. Synovial cell counts and TP concentrations in septic samples can be highly variable.^23^ In addition, the horse had commenced antibiotic treatment prior to sampling, and this may have affected results.

Cytocentrifugation has been used to prepare samples for immunofluorescence, including abdominal and pleural effusions, endotracheal tracheal wash and BAL samples from septic dogs,^42^ and BAL samples from horses with equine asthma.^32,72^ Cytospin preparations are associated with less cell damage compared to direct smears and, by concentrating cells in a small area, the volumes of required reagents and antibodies are minimized. When immunostaining is delayed, freezing of air-dried cytospin smears at −70°C is recommended.^62^ Storage of slides at −20°C has also been reported.^42,52^ Freezing preserves immunoreactivity^62^ and prevents autolysis of cells, which can start to occur after one day of storage at room temperature.^52^ We found that performing immunofluorescence on cytospin preparations that had been stored at −60°C was relatively successful, although some slides had poor cell preservation for unknown reasons. The slide cellularity in 2 samples was too low for NET counts (horse 1, radiocarpal joint; horse 10, PF), despite having similar TNCC to a PF sample from horse 8, which had adequate cells for NET analysis. This could have been the result of loss of sample during preparation, storage, or processing. The use of a desiccant with the stored frozen slides, and ensuring slides have equilibrated to room temperature before removing them from the sealed container to prevent condensation and potential cell rupture, are modifications that might better ensure cell preservation.^20,62^

In the septic fluid samples, it was unclear if NETs were present in appropriate numbers to help control infection or were excessive and contributing to arthritis. In people, higher levels of neutrophil-derived circulating free DNA have been observed in SF from patients with septic arthritis, compared to non-septic samples, and it was suggested that the NETs may contribute to joint lesions including cartilage damage.^45^ NETs have also been identified as playing a role in synovitis and cartilage damage in people with rheumatoid arthritis.^10^ In horses, particularly foals, septic inflammation is thought to contribute to the development of osteochondral lesions^29,74^; however, the involvement of NETs in this process has not been investigated. Abdominal sepsis has also been associated with increased NET markers and lesions. In people with bowel perforation and peritonitis, there was evidence of NETs contributing to intestinal injury and barrier dysfunction.^66^ In a mouse abdominal sepsis model, blocking NET formation reduced mortality.^5^

The NET percentage and neutrophil counts in the septic SF samples were variable and were not correlated, which was an unexpected finding given that there are common signaling pathways and chemoattractants involved in neutrophil chemotaxis and NET release.^13,68,78^ A potent chemoattractant is interleukin-8 (IL-8, CXCL8),^7,68^ which also causes NET release at higher concentrations in vitro.^68^ In addition, NETs themselves can cause neutrophil activation and release of IL-8.^18,57^ Conversely, NET aggregates can degrade chemokines and cytokines, which may reduce recruitment and activation of neutrophils.^27,60^ The reason for the variation in NET percentage and neutrophil count in our study was not clear and likely the result of factors such as the extracellular environment,^37,39,47^ the bacterial species involved,^51,56^ and the neutrophil population.^50^ Additional analysis of the fluid samples, including measuring the pH and IL-8, and evaluating if neutrophils were immature or aged, may have provided further information. We found no clear relationship between the bacterial species and NET percentage; however, sample numbers were too small for a thorough evaluation.

NET staining was similar using the monoclonal and polyclonal Cit-H3 antibodies, and a NET count performed in one of the samples was the same for both antibodies. The similarities in immunofluorescence staining may be because there is more overlap between the antibodies than expected. A human study comparing polyclonal and monoclonal Cit-H3 antibodies demonstrated that many available monoclonal and polyclonal antibodies were unable to differentiate citrullinated from unmodified semi-synthetic nucleosomes, and there was also off-target cross-reactivity with other H3 residues.^69^ It is unknown if this would be the same with equine neutrophils, but nonspecific binding with unmodified histone might explain the weak Cit-H3 fluorescence that we observed with nuclei.

Using separate secondary antibodies linked to different fluorochromes would have been useful to allow visualization of MPO and Cit-H3 in a single merged image and facilitate NET identification. Our plan was to use a Cit-H3 antibody linked to a fluorochrome emitting red fluorescence; however, the manufacturer could not supply the product and there was not a timely alternative. Another issue affecting our study was that the MPO antibody that we used often required a magnification of 1,000× for visualization, which was impractical for NET quantification. Further optimization of the immunofluorescence protocol, a different MPO antibody, or another NET marker, such as an anti-neutrophil elastase antibody,^65^ may have been helpful. Successful visualization of MPO would not only have been useful for helping to identify NETs but also for detecting neutrophils in samples containing mixed cells.^54^ As was observed in a canine study,^42^ it was difficult to differentiate nuclei of neutrophils from other cells in our study, especially when cells were condensed or had a rounded appearance. Therefore, the NET percentage was expressed relative to the total number of nuclei. This is a limitation for samples containing a smaller proportion of neutrophils, as it could underestimate the NET percentage.

Although we successfully identified and quantified NETs using immunofluorescence microscopy and analysis, the methodology would be impractical in a diagnostic laboratory. The process is labor intensive, with only small numbers of slides able to be manually processed simultaneously, and the immunofluorescence can easily fail if there are handling or technical errors. Quantifying NET formation can be challenging, especially if there is variation in cell distribution and if NETs are aggregating. Diluting highly cellular samples with sterile saline would have helped with cell distribution and consistency, and the addition of albumin can assist with cell preservation.^34,42^ Increasing the volume of SF or PF added to the cytospin funnels during the preparation of low cellularity samples may have ensured that sufficient cells were present for NET counting. To account for variation in cellularity between slides, the percentage of cells (nuclei) releasing NETs was counted, similar to a study that reported ratios.^42^ We counted NETs along the edges of cytospin preparations because a previous equine study only detected NETs on the periphery and not in the middle of cytospins. This was suggested as reflecting changes in cell morphology associated with NET production,^72^ and was mostly true for the SF and PF samples in our study; however, small numbers of NETs were present in central parts of some cytospins. The use of semi-automated processing, such as automated slide staining^44^ and counting software programs, which could also measure areas taken up by NETs,^32,54,71^ would make sample preparation and analysis more efficient and minimize variation between runs.

A limitation of our study was the absence of a positive control, to ensure that only NETs were stained. In vitro stimulation of neutrophils to cause NET release^21,32,56^ could have been used as a positive control for staining. The lack of a positive control was a particular limitation for assessment of the monoclonal antibody, given that it has not been used previously on equine samples. The appearance of the NETs, with Cit-H3 fluorescence clearly associated with DAPI-positive DNA, and the similarity in staining to the polyclonal Cit-H3 antibody, were supportive of NETs truly being present. However, further testing would be required to fully assess the efficacy of the monoclonal Cit-H3 antibody for use on equine samples. This would include performing more direct NET count comparisons, using positive control and clinical samples, to confirm that both antibodies detect a similar number of NETs. Given that ours was a clinical study, only a small number of slide replicates were available. Sometimes, one of the paired slides, stained with monoclonal or polyclonal antibody, fluoresced poorly, contained low numbers of cells, or had varied cell preservation. Repeat immunofluorescence staining of suboptimal slides was undertaken on stored slide replicates, and NETs stained with the polyclonal or monoclonal Cit-H3 antibody were visually compared, and they appeared similar. To minimize variation, counts for direct comparison of the 2 antibodies were only carried out using slides stained in the same immunofluorescent run.

Another limitation of our study was the small sample size, particularly the number of useable PF samples; the clinical nature of our research meant that sample numbers and types being submitted to the laboratory during the study period could not be controlled. A NET count could only be performed on one septic PF sample, and therefore the results should be viewed cautiously. In addition, there were low numbers of non-septic samples and no SF and PF samples from clinically healthy horses as true controls. The horses had different histories, including the administration of antimicrobial treatment, and not all had samples available for bacterial culture. This could have affected the classification of samples, and the antimicrobials could also have had an influence on NET release.^9^ Variable collection and processing times might have had an effect on the numbers of NETs observed, given that neutrophils are highly susceptible to activation,^31^ and NETs are also fragile.^6^ There was also the potential for NET release to occur during storage, and to be increased in samples with higher RBC counts; hemoglobin and related molecules,^48^ as well as platelets,^73^ can cause NET formation. Analysis of a small number of low cellularity samples enabled a general assessment of nonspecific cell activation attributable to sample handling or storage, but a more controlled and larger study would be required to investigate this further.

Studies comparing septic and non-septic inflammation, such as from trauma, would be useful, particularly in terms of differences in NET formation associated with cell numbers and types. Further studies might also assess for NETs in larger numbers of fluid samples, including in samples collected sequentially over time. This could provide more information on whether measuring NETs can be helpful for diagnosing septic inflammation, monitoring response to treatment, and for predicting prognosis. It would also be useful to test other NET-specific antibodies, including a monoclonal antibody that detects citrullination of residue R8 on histone H3,^69^ as well as other novel antibodies that may detect NETs at specific stages or those that do not stain with Cit-H3.^71^ Utilizing methods that can analyze larger numbers of samples and provide quantitative data would be of value. Flow cytometry methods have been developed for rapid, high-throughput, and reproducible assessment of NETs^77^; it would be interesting to explore this with equine samples.

## Supplemental Material

sj-pdf-1-vdi-10.1177_10406387231196552 – Supplemental material for Visualizing neutrophil extracellular traps in septic equine synovial and peritoneal fluid samples using immunofluorescence microscopy Click here for additional data file. Supplemental material, sj-pdf-1-vdi-10.1177_10406387231196552 for Visualizing neutrophil extracellular traps in septic equine synovial and peritoneal fluid samples using immunofluorescence microscopy by Emily M. Birckhead, Shubhagata Das, Naomie Tidd, Sharanne L. Raidal and Shane R. Raidal in Journal of Veterinary Diagnostic Investigation
