# Supplementary material for: Visualizing neutrophil extracellular traps in septic equine synovial and peritoneal fluid samples using immunofluorescence microscopy
Source: J Vet Diagn Invest. 2023 Sep 3;35(6):751–60. doi: 10.1177/10406387231196552 (PMC10621558; doi:10.1177/10406387231196552)
Supplement: sj-pdf-1-vdi-10.1177_10406387231196552 – Supplemental material for Visualizing neutrophil extracellular traps in septic equine synovial and peritoneal fluid samples using immunofluorescence microscopy [file sj-pdf-1-vdi-10.1177_10406387231196552.pdf]

Birckhead EM, et al. Visualizing neutrophil extracellular traps in septic equine synovial and peritoneal fluid samples using immunofluorescence microscopy

Supplemental Table 1. Cytospin sample preparation times after synovial or peritoneal fluid collection.

Time post-collection, h	Horse ID
≤8	1, 3, 4, 7–11
12 to <24	2, 5, 6

Supplemental Table 2. Signalment and clinical diagnoses of 11 equine patients who had synovial fluid (horses 1–7), or peritoneal fluid (horses 8–11) collected for cytologic evaluation.

Horse	Age	Sex	Breed	Clinical diagnosis
1	9 y	F	Arab	Multiple limb trauma and lacerations with open CMJ, ICJ, and TCJ
2*	3 y	F	TB	Septic DIPJ and navicular bursitis
3	15 y	CM	QH	Septic MTPJ and fracture of 4th MB
4	1 y	M	TB	Septic FPJ and FTJ
5	15 d	F	TB	Septic TCJ
6	19 d	F	TB	Septic TCJ
7*	18 d	M	SB	Septic bicipital bursitis and humeral osteomyelitis
8	2 y	F	QH	Colic of undefined origin
9*	7 y	F	WB	Septic peritonitis
10	10 y	CM	WB	Suspected sublingual or lingual infection
11	4 y	F	Friesian	Septic peritonitis

CM = castrated male (gelding); CMJ = carpometacarpal joint; d = day(s); DIPJ = distal interphalangeal joint; F = female (mare); FPJ = femoropatellar joint; FTJ = femorotibial joint; ICJ = intercarpal joint; M = male (colt); MB = metatarsal bone; MTPJ = metatarsophalangeal joint; QH = Quarter horse; SB = Standardbred; TB = Thoroughbred; TCJ = tarsocrural joint; WB = Warmblood.

* Patients subjected to euthanasia due to guarded prognosis and/or associated treatment costs; remainder of the patients were discharged from hospital.

Supplemental Table 3. Additional laboratory data from analysis of synovial (horses 1–7) and peritoneal fluid (horses 8–11) samples.

Horse	Differential count	tRBC, × 10^9/L	Bacterial growth
1	NEUT 7%; LMC 74%; LYM 19% (RCJ)	0.1	N
	NEUT 76%; LMC 22%; LYM 2% (TCJ)	4	NA
2	NEUT 97%; LMC 2.5%; LYM 0.5%	0	*Streptococcus dysgalactiae* subsp. *equisimilis*
3	NEUT 67%; LMC 26%; LYM 7%	3	*Staphylococcus aureus*
4	NEUT 96%; LMC 3%; LYM 1% (LFTJ)	920	N
	NEUT 93%; LMC 5%; LYM 2% (MFTJ)	400	N
5	NEUT 96%; LMC 3%; LYM 1%	3.2	*Escherichia coli*
6	NEUT 61%; LMC 33%; LYM 6%	5.6	N
7	NEUT 98.5%; LMC 1%; LYM 0.5%	15	*Escherichia coli*
8	NEUT 80%; LMC 20%	0.1	NA
9	NEUT 94%; LMC 5%; LYM 1%	116	*Escherichia coli* (non-hemolytic)
10	NEUT 85%; LMC 10%; LYM 5%	0	NA
11	NEUT 88%; LMC 4%; LYM 8%	20	Gram negative coccobacilli consistent with *Actinobacillus equuli*

LFTJ = lateral femorotibial joint; LMC = large mononuclear cells; LYM = lymphocytes; MFTJ = medial femorotibial joint; N = no; NA = not applicable (not performed); NEUT = neutrophils; tRBC = total RBC count; RCJ = radiocarpal joint; TCJ = tarsocrural joint.

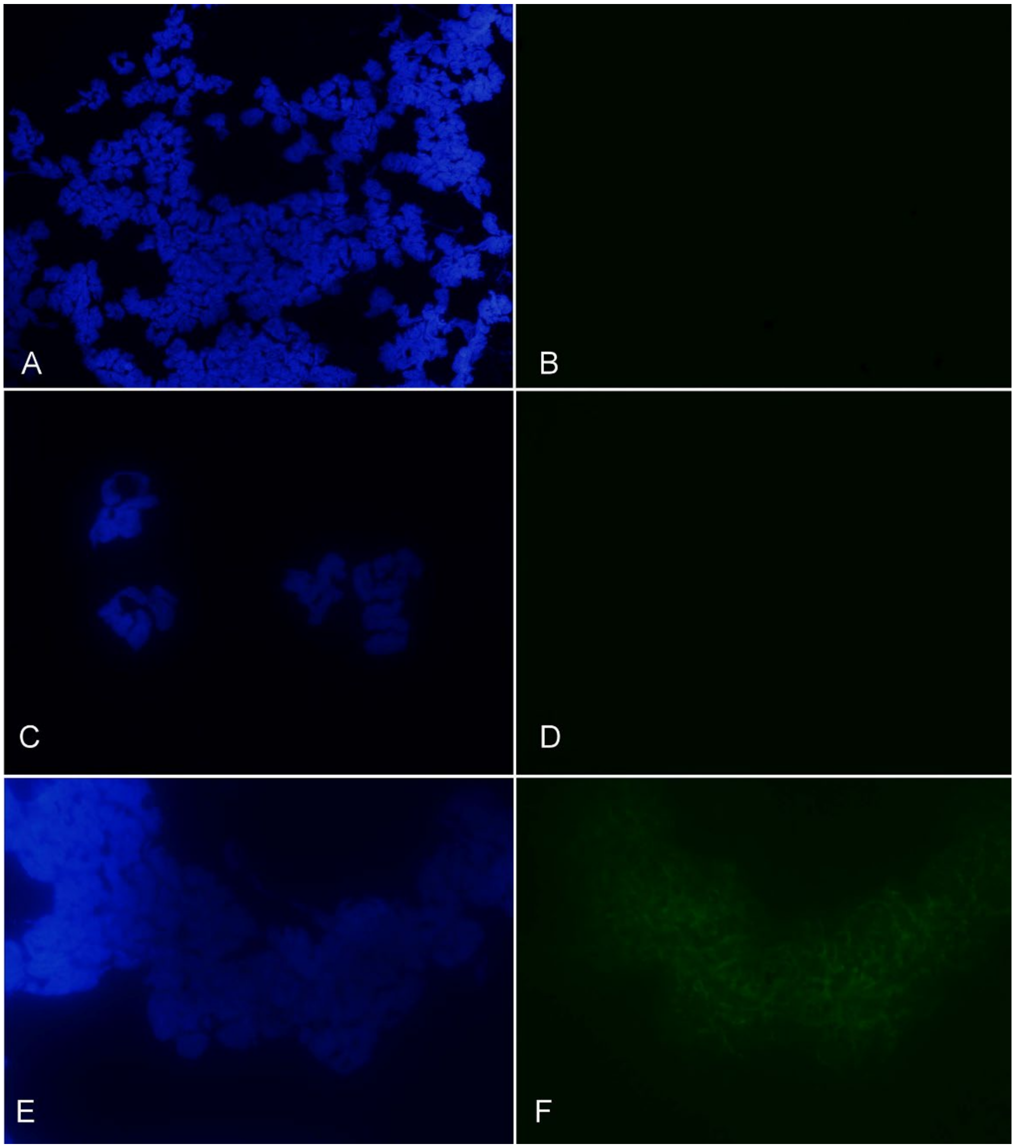

Supplemental Figure 1. To evaluate nonspecific binding of the secondary antibody, synovial fluid cytospin samples were prepared following the normal immunofluorescence methodology, but with exclusion of the primary antibodies. **A, C, E.** The blue DAPI dye was used to identify DNA (nuclei). **B.** Using the FITC filter, no fluorescence was detected at 400× magnification. **D.** At 1,000× magnification, fluorescence was absent to (**F**) sometimes weakly present, with the latter usually associated with dense cell clusters. This nonspecific binding was considered acceptable, given that it was not observable at 400×, the magnification used to count neutrophil extracellular traps (NETs), and it was weak and could not be mistaken for NETs.

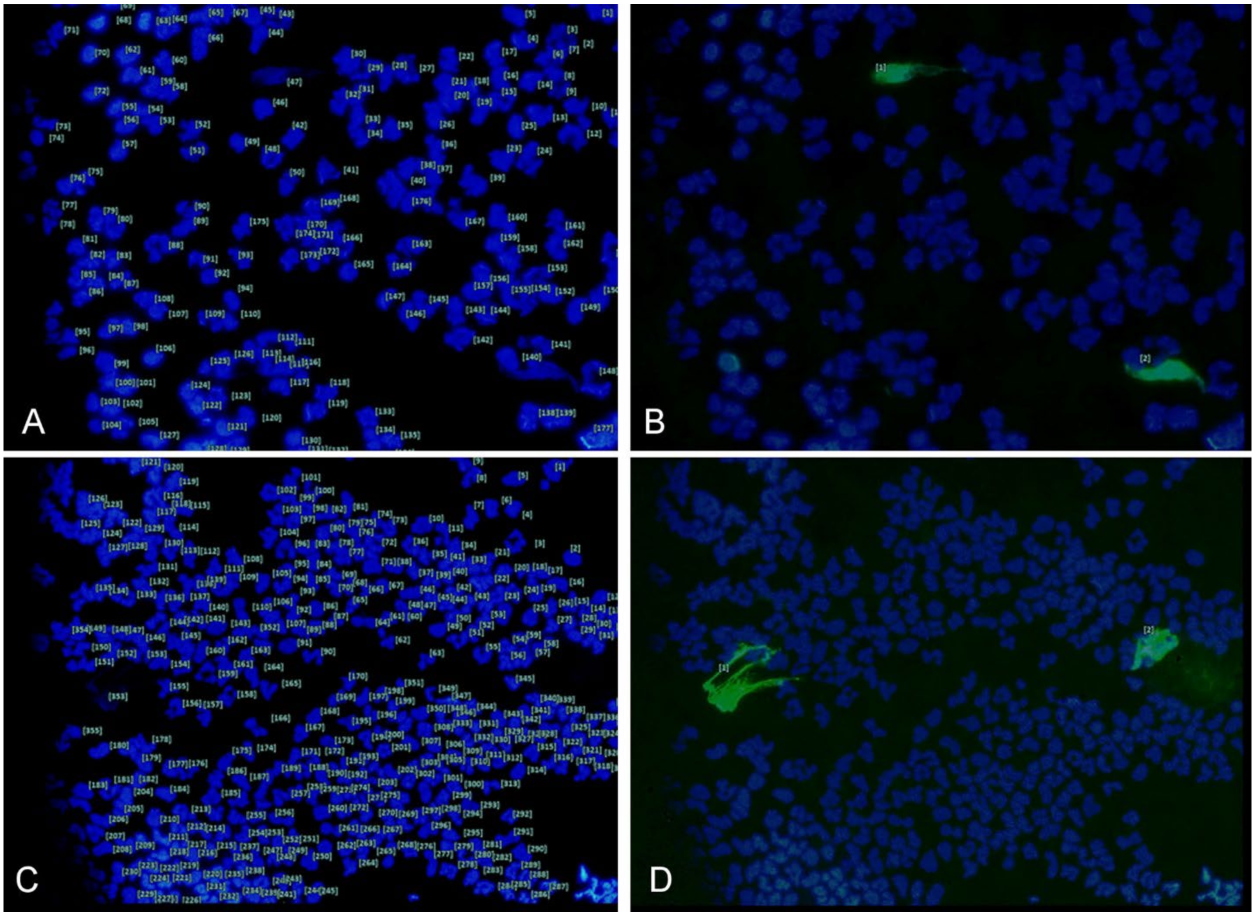

Supplemental Figure 2. Neutrophil extracellular trap (NET) counts were performed using the counting tool in ENVI software (Harris Geospatial Solutions). **A, C.** DAPI (blue) images were used to count cells. **B, D.** FITC (green) images were merged with DAPI images to count NETs. The FITC fluorescence represented citrullinated histone 3 (monoclonal antibody). In these examples, cell numbers were (**A**) 177 and (**C**) 355, and there were 2 NETs present in both merged images. 400×.

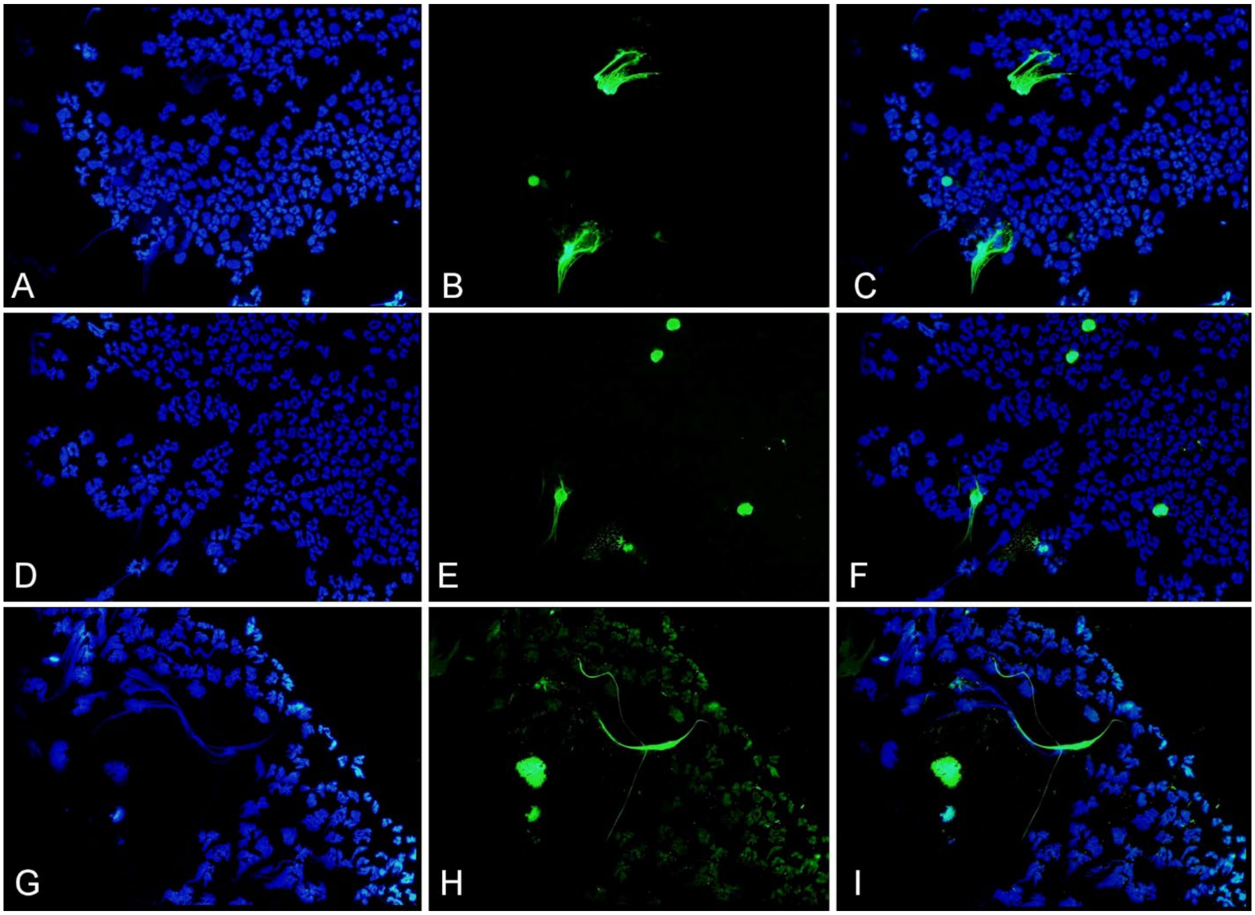

Supplemental Figure 3. Examples of neutrophil extracellular traps (NETs) in cytospins prepared from synovial fluid from horses 3 (A–C) and 7 (D–F), and peritoneal fluid from horse 9 (G–I). **A, D, G.** The blue DAPI filter was used to identify DNA. **B, E, H.** The green FITC filter was used to identify citrullinated histone 3 (Cit-H3). **C, F, I.** NETs were visualized in merged images. Monoclonal (B, E) and polyclonal (H) anti–Cit-H3 antibodies were used. 400×.

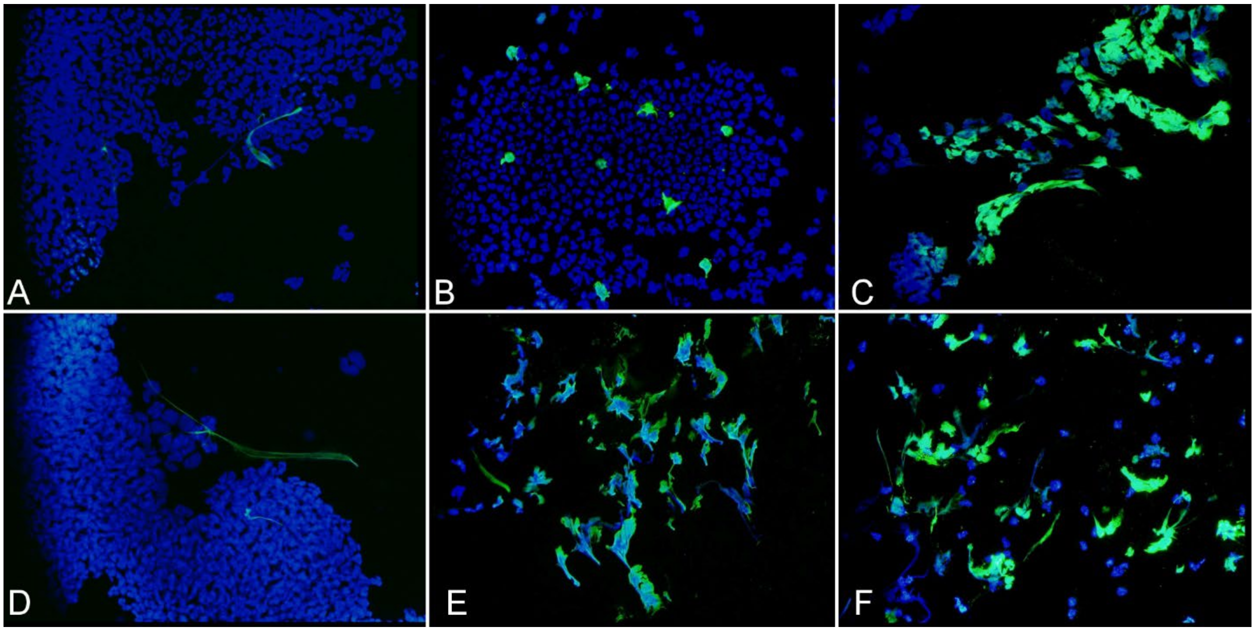

Supplemental Figure 4. Immunofluorescence using antibodies against citrullinated histone 3 (Cit-H3, green), with comparison of monoclonal (**A–C**) and polyclonal (**D–F**) antibodies in paired cytospin smears. DNA (blue) was visualized using the DAPI filter, and all images have been merged. Neutrophil extracellular traps appeared similar in staining with both anti–Cit-H3 antibodies. Samples included synovial fluid from horses 2 (A, D) and 7 (B, E), and peritoneal fluid from horse 9 (C, F). 400×.

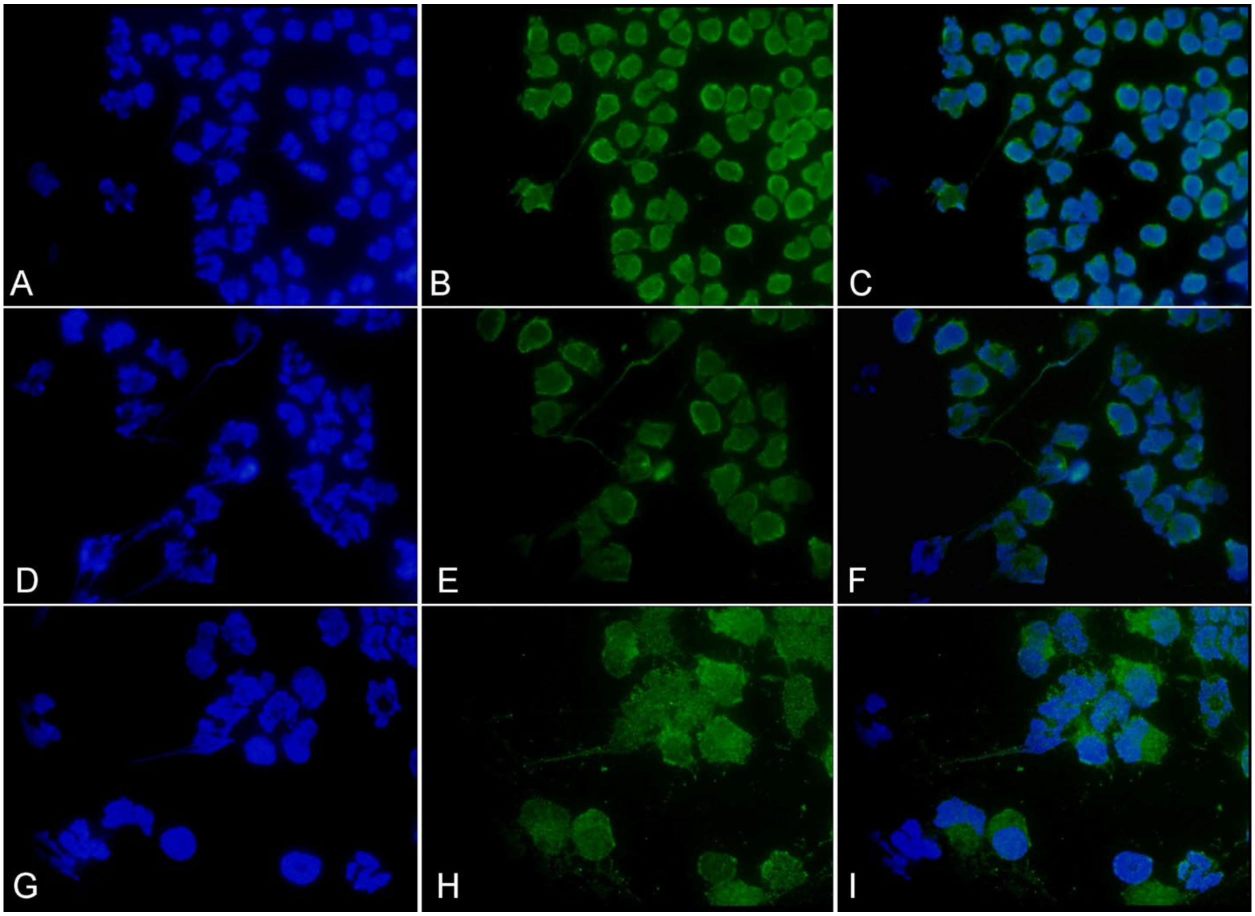

Supplemental Figure 5. Examples of neutrophil extracellular traps (NETs) in tarsocrural synovial fluid cytospins prepared from horses 1 (A–F) and 6 (G–I) using myeloperoxidase (MPO) immunofluorescence. **A, D, G.** The blue DAPI filter was used to identify DNA. **B, E, H.** The green FITC filter was used to identify MPO. **C, F, I.** NETs were visualized in merged images. 1,000×.
